# The COVID-19 pandemic: SARS-CoV-2, childhood hepatitis and monkeypox raise five new questions for the global health research community

**DOI:** 10.7189/jogh.12.01002

**Published:** 2022-05-28

**Authors:** Igor Rudan

**Affiliations:** Centre for Global Health, Usher Institute, The University of Edinburgh, Edinburgh, UK

For decades, there has never been a period in global health that was as unexpected and enigmatic to experts in the field as it is today. Over the past few decades, most of the events that happened in relation to viruses and bacteria in the most developed parts of the world have been largely predictable. Vaccines for the prevention of infectious diseases were effective, as so were the antibiotics available for treatment. It was difficult to get young physicians interested in the epidemiology of infectious diseases.

However, every few years, an unexpected epidemic would appear, caused either by a new or an already known agent – such as SARS and MERS, or Zika and Ebola. However, due to the mode of transmission of these diseases, it was quite feasible for epidemiologists to limit and then suppress them, thanks to the knowledge, experience and rules of treatment that have been documented throughout the history of medicine.

The phenomenon of nosocomial infections caused by specific microbes, which do not threaten people outside hospitals, but manage to persist among many immunocompromised patients within the hospitals, has also come under the scrutiny of epidemiologists. They also grew increasingly concerned about the phenomenon of antibiotic resistance towards antibiotics, as some bacteria began to develop new mutations due to irrational and excessive use of antibiotics in the treatment of people with fever, but also because of their use in livestock and poultry.

However, the work on these global health issues over the past decades has been somewhat predictable. There were fairly clear guidelines and plans laid out for their effective control, and therefore they did not attract much attention from the general public.

The COVID-19 pandemic, however, was quite different. Unexpectedly, a completely new, unknown virus appeared, which managed to gather all the properties that make the epidemiologist's job of suppressing the spread very difficult:

(1) it had a “time window” during which it could spread from the infected to the healthy before the infected developed symptoms;

(2) it not only spread in cough-propelled droplets, but also by aerosol so that it could float imperceptibly in the air, where anyone could inhale it;

(3) it spread atypically easily from person to person, so that in a short time it could lead to a huge number of infected people, which health systems could not cope with, and important areas of human activities would suddenly be left without a large number of workers;

(4) the virus was not limited to humans, but rather spread easily to various animal species, gaining additional opportunities for mutations; then, it could be reintroduced to humans, having acquired new mutations of insufficiently known effect;

(5) it was an RNA virus, not a DNA virus; therefore, its mutation repair mechanisms were weaker and new strains were formed faster. Evolution has directed these strains toward more and more contagious, and toward increasingly successful in avoiding human immune responses, with uncertainty about whether new strains will also lead to a more or less severe clinical picture.

The alpha strain, which originated in the United Kingdom, spread faster than the original Wuhan strain and replaced it while being even more dangerous to human health. The delta strain, which originated in India, spread even faster than alpha and was also more dangerous to health. The omicron strain, which originated in southern Africa, spread even faster than the delta and replaced it, so it now completely covers the planet. Fortunately for us all, in terms of health hazards, omicron mutated in a different direction, so it became significantly less dangerous than the delta, but still remained deadly.

Thanks to the combined effects of the COVID-19 vaccination globally, effective drugs such as Dexamethasone and Paxlovid, and exposure to previous waves among those who were infected, the omicron strain has enabled us to reach the phase of the COVID-19 pandemic where - for the first time – epidemiologists do not expect the next waves to cause as large numbers of deaths globally, as was the case in previous waves. It took the global research community two and a half years to reach that point.

Today, expert estimates agree that in the several major waves of the COVID-19 pandemic so far, at least 15 million people have lost their lives worldwide [1,2]. In the United States alone, the figure of one million deaths has been exceeded [3], which is even worse than the most pessimistic estimates made by experts there at the very beginning of the pandemic [4]. Most deaths have never been documented in official reports of deaths from COVID-19, but are clearly visible in increased mortality compared to expectations based on average mortality from pre-pandemic years [5,6].

After several waves of the COVID-19 pandemic, we have reached the first period in which epidemiologists - thanks to a combination of effective vaccines, available drugs and acquired immunity among the population - feel safer in preventing large numbers of deaths in the coming months. However, they are now faced with five truly unexpected new puzzles, on which they will focus their scientific research in the coming months and years:

(1) The first conundrum, to which no one has an answer yet, is the question if we can now control COVID-19 in the long haul and prevent new waves of this pandemic that would cause further millions of deaths worldwide? Unfortunately, this question cannot be answered today with complete certainty. The virus currently mutates quite quickly and very often. Although we were all lucky that, while acquiring a significantly higher ability to infect, the omicron was significantly less dangerous to human health than the immediately preceding strains, it will continue to cause waves of infection while constantly mutating. No one can know for sure whether, in time, a strain might appear that will be able to bypass our immune protection and be clinically more dangerous. Even the current vaccines only prevent clinical infection and transmission to a varying degree, although they do protect from more severe outcomes much better. Therefore, the absolute global research priority is the development of an advanced vaccine, which will protect us against all possible strains of the SARS-CoV-2 virus. In addition, it would be ideal if such a vaccine could be administered simply, eg, by nasal inhalation, and boost longer-lasting immunity to SARS-CoV-2 in comparison to the current vaccines. Until such a vaccine is developed, any new antiviral drug, such as Paxlovid, will also be of great help [7]. Therefore, despite the current respite, the work for the research community to ensure control over the COVID-19 pandemic is still not complete.

(2) Another riddle, to which no one still has a definite answer, is - why do some people with COVID-19 develop unusual symptoms, lasting for months - the so-called “long COVID” [8]? These people have significantly reduced work capacity and quality of life. How could we recognize such patients, and how to cure their symptoms? Understanding “long COVID” and its effective treatments, or even prevention, will be significant research priorities in global health this year and likely in the future years.

(3) The third open question, which is increasingly troubling, is why has COVID-19 significantly increased the long-term risk of many diseases, such as heart attack and stroke, diabetes, blood clots, psychological disorders and various other already known diseases in those who were infected, especially if they were hospitalized [9,10]? Will this risk remain increased in the long term, or will it return to expected levels over the years? If the risk remains increased for many years, it will eventually lead to a shortening of life expectancy at the level of entire countries. This is another indicator to closely monitor in future years.

(4) The fourth mystery, which has been widely reported in recent weeks, is the unusual epidemic of hepatitis in children. So far, it affected at least 176 children in the UK and more than 500 worldwide [11]. Is it, in some way, related to the COVID-19 pandemic? Is it caused by an adenovirus, or perhaps SARS-CoV-2, or their combined action? An alternative hypothesis is that this could just be an unusual, difficult-to-explain, transient epidemic of a virus, which we were able to record only because of the significantly increased interest in infectious diseases and their control during the COVID-19 pandemic. No one has a definite answer to that question yet.

(5) The fifth conundrum is the appearance of monkeypox in an increasing number of cities in developed countries outside Africa, among people who may not all be clearly interconnected, nor have they travelled to Africa. So far, more than 100 cases have been confirmed in Europe, North America and Australia [12]. Fortunately, the first genomic analyses have shown that it is the milder of the two strains circulating in Africa, and they seem to imply a common origin, at least so far [13]. The West African strain, which seems to be implicated, is quite mild, while the one from Central Africa is much more dangerous. Also, the spread of this disease is expected to be quite different from COVID-19 and it should be more easily containable, while the stockpiled smallpox vaccines may also be deployed to assist in breaking the chains of transmission. The question remains - does this epidemic have anything to do with COVID-19, or not at all? We'll gradually find out.

Since the end of March 2020, there has been an extremely high degree of consensus in the scientific community in interpreting all new information on COVID-19. The countries that knew how to use this information well have kept mortality from COVID-19 to a minimum and also protected their economies from a larger downturn, while requiring varying degrees of changes in lifestyle from their citizens.

Thanks to the global health research community, and the exchange of information among its experts, we have gained the necessary knowledge that currently reduces the impact of the COVID-19 pandemic. We now have accurate estimates of the effectiveness of various measures that can mitigate the spread of the virus, we know the benefits of dexamethasone, the effectiveness of various vaccines against various strains of the virus, as well as the effects of early administration of Paxlovid. With all these interventions, the risk of dying from COVID-19 disease is now generally reduced by at least ten compared to the onset of a pandemic – in older age groups even more [14].

In two and a half years, the global health research community and the pharmaceutical and biotechnological industries managed to protect many people from dying from COVID-19 and finally bring large parts of the world back to a pre-pandemic lifestyle. What is especially gratifying is knowing that modern science has all the tools available to also provide many answers to all these five emerging research questions in the coming months and that it will most likely succeed in answering them, too.

## References

[1] World Health Organization. 14.9 million excess deaths associated with the COVID-19 pandemic in 2020 and 2021. News release, 5 May 2022. Available from: https://www.who.int/news/item/05-05-2022-14.9-million-excess-deaths-were-associated-with-the-covid-19-pandemic-in-2020-and-2021. Accessed: May 21, 2022.

[2] Grimley N, Cornish J, Stylianou N. Covid: World’s true pandemic death toll nearly 15 million, says WHO. BBC News, 5 May 2022. Available from: https://www.bbc.co.uk/news/health-61327778. Accessed: May 21, 2022.

[3] Donovan DUS. officially surpasses 1 million covid-19 deaths. Johns Hopkins University of Medicine Coronavirus Resource Center, 16 May 2022. Available from: https://coronavirus.jhu.edu/from-our-experts/u-s-officially-surpasses-1-million-covid-19-deaths. Accessed: May 21, 2022.

[4] Coronavirus: White House models predict up to 240,000 fatalities. Financial Times, 31 May 2020. Available from: https://www.ft.com/content/1581cbdb-c47e-3073-b629-017397a981e5; Accessed: May 21, 2022.

[5] Tracking COVID-19 excess deaths across countries. Available from: https://www.economist.com/graphic-detail/coronavirus-excess-deaths-tracker. Accessed: 21 April 2022.

[6] Excess Death” Data Point to Pandemic’s True Toll. Available from: https://www.cdc.gov/coronavirus/2019-ncov/cdcresponse/accomplishments/excess-death-data.html. Accessed: 21 May 2022.

[7] European Medicines AgencyPaxlovid. Available from: https://www.ema.europa.eu/en/medicines/human/EPAR/paxlovid; Accessed: 21 May 2022.

[8] Davis HE Assaf GS McCorkell L Wei H Low RJ Re’em Y Characterizing long COVID in an international cohort: 7 months of symptoms and their impact. EClinicalMedicine. 2021;38:101019. 10.1016/j.eclinm.2021.101019 34308300 PMC8280690

[9] Watson C Diabetes risk rises after COVID, massive study finds. Nature. 2022. Online ahead of print. 10.1038/d41586-022-00912-y 35361939

[10] Sidik SM Heart-disease risk soars after COVID — even with a mild case. Nature. 2022. Online ahead of print. 10.1038/d41586-022-00403-0 35145295

[11] Binnicker M. Adenovirus or Covid-19: What is behind the outbreak in child hepatitis cases? Available from: https://www.forbes.com/sites/coronavirusfrontlines/2022/05/19/adenovirus-or-covid-19-what-is-behind-the-outbreak-in-child-hepatitis-cases. Accessed: 21 May 2022.

[12] World Health Organization. Monkeypox. News release, 19 May 2022. Available from: https://www.who.int/news-room/fact-sheets/detail/monkeypox. Accessed: 21 May 2022.

[13] Kozlov M Monkeypox goes global: why scientists are on alert. Nature. 2022. Online ahead of print. 10.1038/d41586-022-01421-8 35595996

[14] Burn-Murdoch J, Barnes O. Vaccines and omicron mean COVID now less deadly than flu in England. Financial Times, 10 Mar 2022. Available from: https://www.ft.com/content/e26c93a0-90e7-4dec-a796-3e25e94bc59b. Accessed: 21 May 2022.

